# Case Report: Craniotomy and radiotherapy for multiple hemorrhagic cerebral lesions from resected cardiac myxoma

**DOI:** 10.3389/fonc.2026.1856684

**Published:** 2026-07-22

**Authors:** Hui Li, Peng Zhang, Haoran Chen, Xiaoyan Gao, Qiushi Zhang, Juan Li, Jing Tong

**Affiliations:** 1 Department of Neurosurgery, The Fourth Hospital of Hebei Medical University, Shijiazhuang, Hebei, China; 2 Department of Radiation Oncology, The Fourth Hospital of Hebei Medical University, Shijiazhuang, Hebei, China

**Keywords:** cardiac myxoma, craniotomy, intracranial lesions, prognosis, radiation therapy

## Abstract

This report elaborates on a unique case of multiple cerebral lesions from completely resected atrial myxoma which was treated with craniotomy and radiotherapy. A 56-year-old female presented with progressive headache, left limb weakness and low-grade fever for over 20 days. She had undergone total resection of left atrial myxoma nine months before. MRI revealed multiple hemorrhagic lesions in both cerebral hemispheres and left cerebellum. The symptomatic lesion in the right parietal lobe was resected under neuronavigation, ultrasound and electrophysiological monitoring. Pathology confirmed myxoma seeding. Whole-brain radiotherapy (30 Gy in 15 fractions) was administered for the remaining multiple lesions. During 14 months of follow-up, the patient’s left limb muscle strength improved from grade V^-^ to grade V, MRI showed no disease progression, and Karnofsky Performance Status (KPS) increased from 70 preoperatively to 90. One epileptic seizure was well controlled with medication.

## 1 Introduction

Myxoma is the most common primary benign cardiac tumor in adults, accounting for the majority of cardiac tumors. While it can occur across a wide age range, it is most frequently diagnosed in individuals between 30 and 60 years old, with a higher prevalence in women (1). Although it primarily presents as a benign tumor, a myxoma carries a high risk of embolism due to its specific location and tendency to detach (2). It is most commonly found in the central nervous system (3). In the central nervous system, it often manifests as cerebral infarction (4), intracerebral hemorrhage, cerebral aneurysms (5), and, rarely, brain parenchymal lesions. Due to the low incidence of myxoma involvement to the brain, there is a lack of standardized treatment protocols (6). This report elaborates on a unique case of multiple cerebral lesions from completely resected atrial myxoma which was treated with craniotomy and radiotherapy.

## 2 Case description

### 2.1 Patient information

A 56-year-old female was admitted with progressive headache, left limb weakness and low-grade fever for more than 20 days. Nine months prior, she had been diagnosed with left atrial myxoma and underwent total resection. She had no other significant medical, family or psychosocial history.

### 2.2 Clinical findings

Neurological examination showed clear consciousness, fluent speech, left limb muscle strength grade V^-^, right limb muscle strength grade V. Cardiac ultrasound confirmed no residual cardiac lesions.

### 2.3 Timeline

- 9 months before admission: Total resection of left atrial myxoma.
- 20 days before admission: Onset of headache, left limb weakness and low-grade fever.
- Admission: Brain MRI showed multiple intracranial lesions.
- During hospitalization: Resection of right parietal lesion.
- Post-operation: Whole-brain radiotherapy (2 Gy/fraction, total 30 Gy).
- 2 months after radiotherapy: Follow-up MRI.
- 14 months after treatment: Last follow-up.

### 2.4 Diagnostic assessment

**Figure 1:**
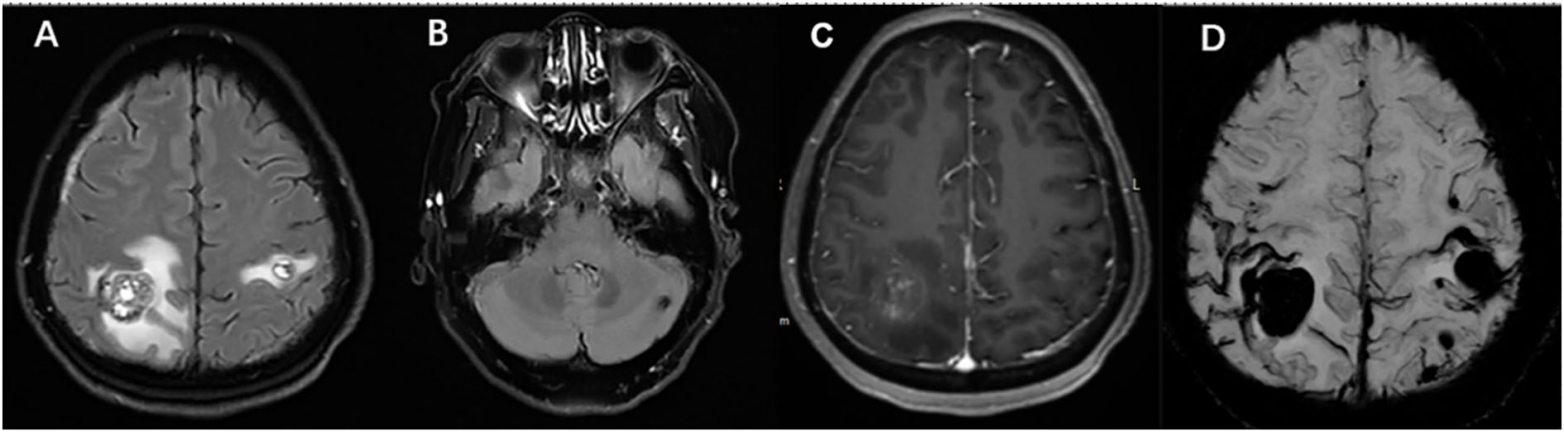
Preoperative MRI images of this patient with atrial myxoma resection surgery. **(A, B)** Preoperative FLAIR images show multiple nodular shadows in the bilateral parietal lobes and left cerebellar hemisphere; **(C)** T1-weighted contrast-enhanced imaging reveals heterogeneous enhancement in the parietal lobe; **(D)** Susceptibility-weighted imaging (SWI) demonstrates multiple hemorrhagic lesions in the bilateral parietal lobes.

Preoperative brain MRI was performed using a 3.0-T scanner with standard sequences, including T1-weighted, T2-weighted, fluid-attenuated inversion recovery (FLAIR), contrast-enhanced T1-weighted, and susceptibility-weighted imaging (SWI). The images revealed multiple intraparenchymal lesions in bilateral cerebral hemispheres and the left cerebellum. The lesions exhibited heterogeneous enhancement on T1-weighted contrast-enhanced sequences and were surrounded by perilesional hyperintense edema on FLAIR. SWI demonstrated hypointense signal areas consistent with hemorrhagic components and hemosiderin deposition (**Figures 1A–D**). Tumor markers and systemic screening excluded other malignancies. The diagnosis was highly suspected as multiple cerebral lesions from resected cardiac myxoma. Differential diagnosis included hemorrhagic metastatic tumors (melanoma, choriocarcinoma, and thyroid cancer), cerebral cavernous malformation, and infective emboli from bacterial endocarditis. The patient’s history of resected cardiac myxoma, together with the multifocal hemorrhagic appearance on susceptibility-weighted imaging (SWI), favored myxoma-related lesions.

### 2.5 Therapeutic intervention

**Figure 2:**
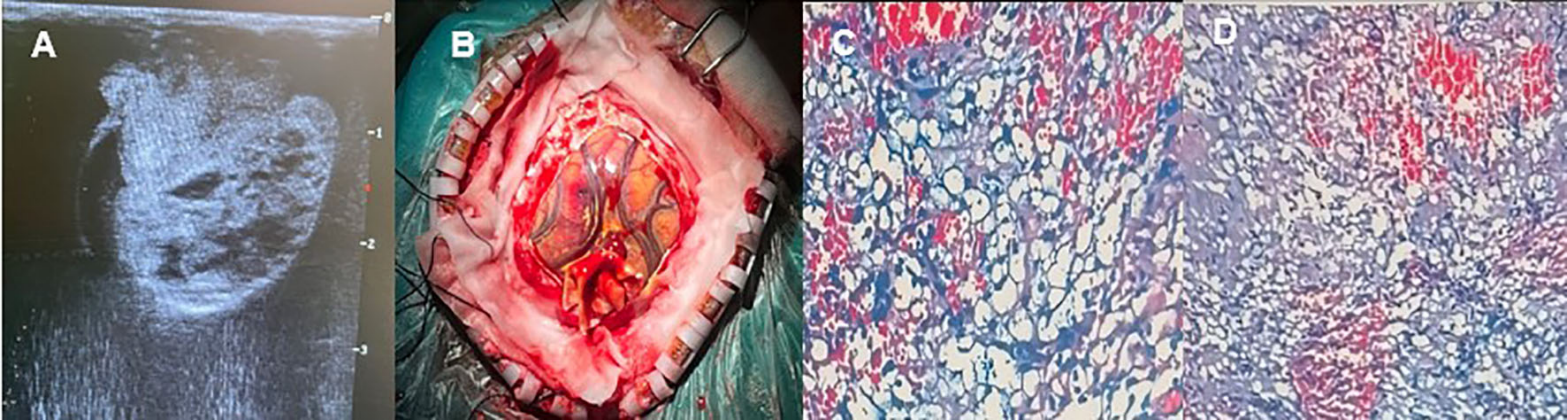
Intraoperative ultrasound image, tumor and pathology pictures. **(A)** Intraoperative Doppler ultrasound image; **(B)** Intraoperative figure shows hemosiderin deposition on the brain surface and the lesion was closely adherent to the dura mater; **(C)** Low magnification HE staining (×10): Multifocal intraparenchymal hemorrhage, diffuse brain edema, proliferative small blood vessels and scattered inflammatory cells; focal pale loose stroma is visible. **(D)** High magnification HE staining (×40): Spindle-shaped stromal cells scattered within pale eosinophilic loose matrix, with interstitial fibrous hyperplasia and focal residual hemorrhage pigment deposition. No widespread atypical mitoses or massive malignant necrosis are identified in this field.

A right parietal craniotomy was performed under general anesthesia. Neuronavigation was used for preoperative planning and intraoperative lesion localization. Intraoperative ultrasound was utilized to confirm the lesion margins and guide the resection. Motor evoked potentials (MEPs) and somatosensory evoked potentials (SSEPs) were continuously monitored throughout the procedure to assess the integrity of the corticospinal tracts and sensory pathways. The lesion was completely resected under microscopic visualization. The involved dura mater was excised and sent for histopathological examination. Bipolar coagulation and absorbable gelatin sponge were used for hemostasis (**Figure 2A**). Surgical indication: The right parietal lesion was selected for resection because it was the largest and caused progressive left-sided weakness. Intraoperatively, the tumor was yellowish-red, soft, 3.5 cm × 2.5 cm × 2.5 cm, moderately vascular and adherent to dura (**Figure 2B**). Postoperative management included antiedema therapy (mannitol) and sodium valproate for seizure prophylaxis. Postoperative pathology confirmed myxoma seeding (**Figures 2C, D**). Microscopic examination revealed spindle or stellate cells with small nuclei in a myxoid background, with scattered hemosiderin deposition and focal hemorrhagic necrosis. Immunohistochemical studies were not performed due to limited tissue availability, which represents a limitation of the histopathological analysis. Rationale for adjuvant radiotherapy: The patient presented with multiple bilateral hemorrhagic lesions affecting both cerebral hemispheres and the cerebellum. Stereotactic radiosurgery (SRS) was not indicated, as deep-seated lesions carry a high risk of radiation-induced necrosis, and it is unfeasible to target all lesions. Other treatment alternatives, such as systemic chemotherapy or conservative management, were not considered appropriate in this case. Chemotherapy (e.g., temozolomide) has been reported in a limited number of cases, but its efficacy remains uncertain and it was not prioritized given the presence of multiple symptomatic lesions with hemorrhagic features. Conservative observation was also deemed unsuitable because the patient presented with progressive neurological deficits, which indicated a risk of further clinical deterioration without active intervention. Therefore, a total dose of 30 Gy whole-brain radiotherapy (WBRT) administered in 15 fractions was adopted for adjuvant treatment. Recent literature including two case reports has described the successful use of WBRT in cases of multifocal hemorrhagic intracranial myxoma (7, 8). Both studies suggested that whole-brain radiotherapy (WBRT) may provide long-term disease control in selected patients.

### 2.6 Follow-up and outcomes

**Figure 3:**
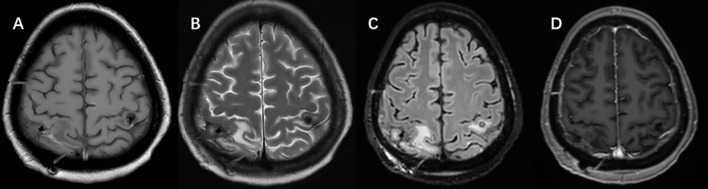
Head MRI images two months after radiotherapy. No changes were observed in the remaining lesions. **(A)** (T1-weighted image), **(B)** (T2-weighted image), **(C)** (FLAIR image) and **(D)** (T1-weighted contrast-enhanced image).

The patient had a good recovery, and the weakness of the left limb was relieved, with the muscle strength improved from grade V^-^ to grade V. Follow-up MRI performed two months after completion of radiotherapy demonstrated no significant interval change in the size or enhancement pattern of the residual lesions, and no new lesions were detected (**Figures 3A–D**). In the 14-month follow-up, she suffered one epileptic episode, and symptoms were fully controlled via antiepileptic medication. The patient’s Karnofsky Performance Status (KPS) increased from 70 preoperatively to 90 at the latest follow-up. No additional adverse events, including cognitive decline and new-onset neurological deficits, were noted.

## 3 Discussion

### 3.1 Mechanisms of intracranial dissemination

Although the term “metastasis” is often used clinically, cardiac myxoma is histologically benign. The currently accepted mechanism is embolic tumor implantation rather than true metastatic disease. Tumor fragments may detach from the primary atrial tumor and travel through the bloodstream, eventually implanting into cerebral vessel walls or directly invading brain parenchyma (6, 9). The hemorrhagic tendency of these lesions is likely attributable to tumor cell invasion of vessel walls and secretion of matrix metalloproteinases (MMPs), which increase vascular fragility (2, 6).

### 3.2 Delayed presentation after complete cardiac resection

Even after complete resection of the primary cardiac tumor, pre-existing microscopic emboli may proliferate gradually and result in the delayed onset of intracranial lesions. In this patient, intracranial lesions emerged 14 months following cardiac surgery, a finding consistent with prior literature (6, 9). This phenomenon underscores the need for long-term neurological surveillance in patients with a history of cardiac myxoma, as recommended by prior studies (6).

### 3.3 Treatment strategies and comparison with published cases

Existing evidence suggests that myxoma cells can enter the cerebral vasculature via the bloodstream, causing vessel wall damage or direct brain invasion, ultimately forming metastatic foci (6). These foci are often accompanied by hemorrhage and perilesional edema, manifesting as heterogeneous enhancement or mass effect on imaging (10). For patients with brain parenchymal lesions, clinical manifestations are generally determined by the location and size of the lesions, which may include headache, epilepsy and focal neurological deficits. Characteristic cranial MRI manifestations consist of multiple hemorrhagic lesions presenting heterogeneous enhancement on T1-weighted sequences, accompanied by surrounding hyperintense edema on FLAIR. Susceptibility-weighted imaging (SWI) is capable of identifying hemosiderin deposits (6, 10, 11).

For left atrial myxoma, early complete resection of the primary tumor is the current first-line treatment to prevent fatal or disabling outcomes. In patients with multiple, bilateral, hemorrhagic lesions not amenable to focal therapy, several recent case reports have confirmed that whole-brain radiotherapy (WBRT) can achieve favorable outcomes (7, 8). In the present case, the patient presented with progressive neurological symptoms and multiple intracranial lesions from atrial myxoma; precise surgical resection combined with postoperative WBRT was associated with a favorable clinical outcome in this patient. During craniotomy, multimodal intraoperative guidance techniques, such as neuroelectrophysiological monitoring, neuronavigation and intraoperative ultrasound, can substantially lower surgical risks and optimally preserve neurological function. Moreover, even after resecting brain lesions resulting from atrial myxoma, epilepsy remains a major clinical issue, calling for sustained attention and standardized management from clinicians.

Treatment options for intracranial myxoma lesions include surgical resection, stereotactic radiosurgery (SRS), WBRT, and chemotherapy (temozolomide) (9, 12). Symptomatic or large lesions accompanied by mass effect warrant surgical resection (9). Small, non-hemorrhagic, countable lesions may be treated with SRS, as reported by Ma et al. using Gamma Knife radiosurgery (9). In contrast, multiple, diffuse, or hemorrhagic lesions not amenable to focal therapy are generally treated with WBRT (7, 8). Unlike the case reported by Ma et al. (9), in which Gamma Knife radiosurgery was feasible for a limited number of small non-hemorrhagic lesions, our patient presented with a high number of hemorrhagic lesions distributed diffusely across both hemispheres, making SRS impractical.

Recent clinical case reports have verified the therapeutic efficacy of WBRT in treating multifocal hemorrhagic intracranial myxoma lesions. Khong et al. (7) reported a 49-year-old woman with multiple hemorrhagic brain lesions from atrial myxoma who achieved complete remission after WBRT and remained disease-free for seven years, suggesting that WBRT may provide long-term control of hemorrhagic brain lesions from cardiac myxoma in selected cases. Basu et al. (8) described a 56-year-old female patient who developed multiple hemorrhagic cerebral lesions three months following resection of a cardiac myxoma. After WBRT administered at a total dose of 20 Gy delivered over five fractions, the patient’s condition remained stable without newly emerging lesions at the 4-year follow-up; her neurological function recovered completely. This case illustrates the potential role of radiotherapy for the treatment of cerebral lesions secondary to left atrial myxoma. In contrast, Ghodasara et al. (6) reported a case without adjuvant radiotherapy, underscoring the potential benefit of WBRT in preventing disease progression.

Compared with previously reported cases without adjuvant radiotherapy (6), our patient achieved 14 months of disease stability with only one controllable seizure, suggesting that adjuvant WBRT may improve clinical outcomes in selected patients with multifocal intracranial myxoma lesions. Similar to the cases documented by Khong et al (7) and Basu et al (8), our patient received adjuvant whole-brain radiotherapy (WBRT) following surgical resection of the symptomatic lesion, reflecting a consistent treatment approach for multifocal hemorrhagic disease. In contrast to the case reported by Ma et al. (9), where Gamma Knife radiosurgery was used for a limited number of small non-hemorrhagic lesions, our patient required WBRT due to the high lesion burden and hemorrhagic features.

### 3.4 Strengths and limitations

The strengths of this case report lie in its documentation of a rare presentation featuring multiple cerebral lesions secondary to completely resected atrial myxoma; the patient received craniotomy combined with radiotherapy, with comprehensive supporting evidence including pathological confirmation, targeted surgical resection guided by multimodal intraoperative navigation, and 14-month mid-to-long-term postoperative follow-up data. Limitations include the single-case nature, lack of follow-up beyond 14 months, and the absence of immunohistochemical analysis for the pathological specimen. Therefore, our findings should be interpreted as hypothesis-generating rather than definitive.

## 4 Patient perspective

The patient provided written informed consent for the publication of this report. She reported that she initially sought medical attention because of progressive headache and left-sided weakness that interfered with her daily activities. She understood the diagnosis of multiple intracranial lesions from cardiac myxoma and the rationale for the combined surgical and radiotherapeutic approach. She expressed satisfaction with the improvement in her neurological symptoms following treatment; she was able to regain independence in activities of daily living and has remained on regular follow-up. She was also aware of the potential risks and benefits of the treatment and agreed to long-term follow-up. She reported good tolerance to both the surgical procedure and the radiotherapy, with only mild fatigue during the radiation course.

## Data Availability

The original contributions presented in the study are included in the article/supplementary material. Further inquiries can be directed to the corresponding author.
